# Boosting natural history research via metagenomic clean-up of crowdsourced feces

**DOI:** 10.1371/journal.pbio.3000517

**Published:** 2019-11-07

**Authors:** Amrita Srivathsan, Niranjan Nagarajan, Rudolf Meier

**Affiliations:** 1 Department of Biological Sciences, National University of Singapore, Singapore; 2 Computational and Systems Biology, Genome Institute of Singapore, Singapore; 3 School of Medicine, National University of Singapore, Singapore; 4 Tropical Marine Science Institute, National University of Singapore, Singapore

## Abstract

Biodiversity is in crisis due to habitat destruction and climate change. The conservation of many noncharismatic species is hampered by the lack of data. Yet, natural history research—a major source of information on noncharismatic species—is in decline. We here suggest a remedy for many mammal species, i.e., metagenomic clean-up of fecal samples that are “crowdsourced” during routine field surveys. Based on literature data, we estimate that this approach could yield natural history information for circa 1,000 species within a decade. Metagenomic analysis would simultaneously yield natural history data on diet and gut parasites while enhancing our understanding of host genetics, gut microbiome, and the functional interactions between traditional and new natural history data. We document the power of this approach by carrying out a “metagenomic clean-up” on fecal samples collected during a single night of small mammal trapping in one of Alfred Wallace’s favorite collecting sites.

## Abstract 2

Natural history research is in crisis and non-charismatic species are increasingly ignored; this Community Page article argues and demonstrates that shotgun sequencing of serendipitously obtained faecal samples could reverse this trend for 1000 mammal species within 10 years.

## Introduction

Natural history research has been in steep decline over the past few decades [1–3]. This is worrying because natural history data are important for the informed management of endangered species based on species-specific traits [3]. The crisis in natural history research and education has been well documented [1,4,5]. It constitutes a particularly serious problem for rare or rarely studied, noncharismatic species. It is these species that have historically been the major beneficiary of the kind of incidental observations that are published in natural history papers. In contrast, most of today’s studies focus on large and high-profile species [6–11], which are given funding priority and preferred by high-impact journals [10,12].

Here, we argue that one way to boost research on neglected species is enriching incidental natural history observations by obtaining “forensic” DNA evidence using new sequencing technologies. For example, traditional pollinator studies can benefit from sequencing flowers in order to detect entire pollinator communities [13], predator–prey studies focusing on spiders can obtain additional data by sequencing spider webs [14], sequencing the gut content of carcasses can reveal much biology [15], and the results of mammal-trapping studies can be enhanced by sequencing fecal DNA. Fecal samples are routinely obtained during fieldwork, but they are currently mostly discarded, although they can yield a rich amount of natural history data. We here argue that the metagenomic evaluation of such samples can enhance traditional natural history observations and make it more likely that they are published.

Natural history research aims to document the flora and fauna and their interactions in a habitat. Such research is heavily dependent on incidental observations by field researchers and citizen scientists [16]. For example, the 11-year expedition of Henry Walter Bates to Amazonia not only led to the discovery of new species, descriptions of the diversity, and deepened our understanding of mimicry, but his *The Naturalist on the River Amazons* is also full of observations on species’ behavior and interactions. This includes observations on the pinktoe tarantula (*Avicularia avicularia*) eating a small finch, scarlet and blue macaws feeding on a bacaba palm, the hyacinthine macaw’s ability to feed on “excessively hard nut (of the fruit of the Tucuma palm) which is crushed into pulp by the powerful beak of the bird,” or on a tamarin which “generally fed on sweet fruits, such as the banana but it is also fond of insects; especially soft-bodied spiders and grasshoppers, which it will snap up with eagerness when within reach” [17]. Similarly, Alfred Russel Wallace’s work in Southeast Asia described in *The Malay Archipelago* [18] includes numerous incidental observations such as the feeding behavior of colugos in Singapore and Borneo, orangutans in Borneo, babirusa in Sulawesi, and fruit pigeons feeding on nutmeg in Banda. Occasionally, the field observations were complemented by information from captive animals (e.g., birds of paradise feeding), but many such observations would today be considered unworthy of publication. Yet, this information continues to inspire follow-up research.

Akin to a naturalist’s incidental field observations, a small number of fecal samples for noncharismatic mammal species may initially appear to be of limited value, but they can be very informative once they are fully evaluated. This is because fecal samples contain information on diet, genetics of the host, gut parasites, microbiome [19–22], and even hormones (e.g., cortisol [23]); i.e., these samples are inherently data-rich and multidimensional. There is a long tradition of unlocking some of this information through morphological study of diet remains, but this only yields information on one dimension and is only feasible for species with incomplete digestion. These limitations can now be overcome by sequencing fecal DNA using high-throughput sequencing technologies. Shotgun sequencing yields not only species-level information on diet but also information relevant to health (e.g., via gut parasites, microbiome) and host genetics [19,20,24]. Fecal metagenomics is also attractive because it requires minimal wet lab work; only the DNA needs to be extracted and sent for sequencing. Very little experience and equipment are needed because new specialized extraction tools are now available (e.g., Terralyzer).

The simplicity of the molecular work renders shotgun metagenomics particularly attractive for field researchers, but there are alternatives that are cheaper but require more lab work. For example, “metabarcoding” can be used for characterizing a single dimension of the sample (e.g., diet or microbiome [25,26]) by amplifying one or several genes that are informative with regard to the targeted dimension. The downside is that the amplification of genes often requires optimization, which is only worthwhile when a large number of samples for the same or closely related species are scrutinized for the same information (e.g., diet) [26]. Targeted approaches are thus arguably less attractive to natural history researchers, who tend to be interested in a diversity of traits and want to discover the unexpected in a small number of samples for the same species. For example, metagenomics would readily reveal that chimps hunt monkeys, tortoises [27], and probably many other animals, whereas repeated diet characterization with plant genes would fail to yield such novel insights. This raises the question of why shotgun metagenomics has only recently been used for unlocking the information contained in fecal DNA. Presumably, the main concerns have been high cost and the complexities of the bioinformatic analysis. However, cost of sequencing is declining fast, and new user-friendly bioinformatics tools are now available [28,29]. They can be used to rapidly obtain species-level identifications despite the need for processing millions of sequences. It thus appears likely that metagenomics will become the method of choice for the study of those species for which few samples are available.

## Mammalian field work: Species coverage over the last few decades

**Fig 1:**
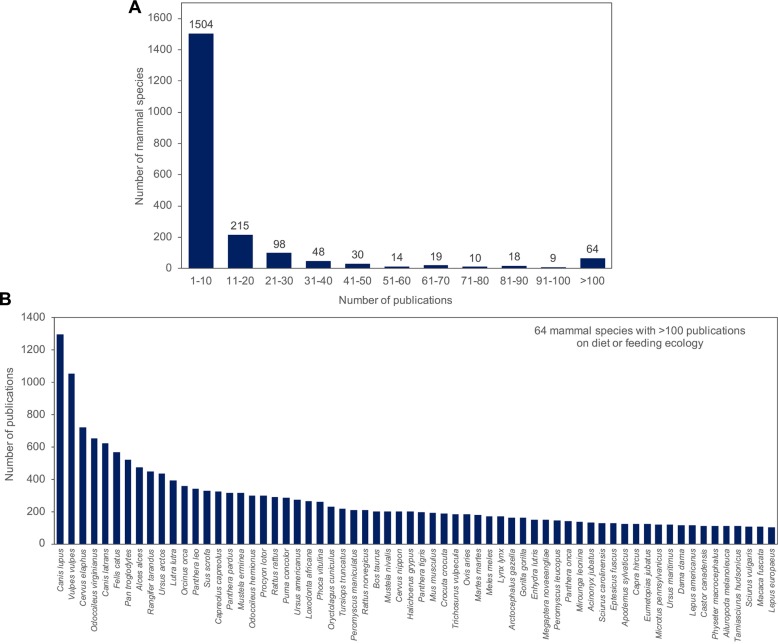
(A) Number of records related to diet/feeding behavior for mammalian species. (B) Species with >100 records. The data underlying this figure are in S1 Data.

But for how many species could we obtain rich natural history data if a substantial proportion of mammalian field surveys were to collect fecal samples? We here use a literature survey to estimate the number of mammal species that have been encountered in the field over the past decades. Estimating this number is difficult because many routine mammalian surveys are not published and/or omit information on off-target, noncharismatic species that were only occasionally encountered. However, one can obtain lower-bound estimates using the published literature catalogued in *Zoological Record* (electronic version: 1978–2018). We thus queried title, abstract, and all other associated field tags for 305,785 mammal publications (authors, publication dates, descriptors, systematics, etc.). We then used the mammal checklist of 6,399 extant species by Burgin and colleagues (2018) [30] to identify 226,021 records that contain recognized species name(s) for extant species. We found that most mammal species were mentioned in the literature (5,860 of 6,399 extant mammals). We then identified those likely to have been encountered in the field. For this, we carried out multiple searches with different logical connectors related, for example, to diet or field work (see Table A in S1 Text). Afterwards, we used a random subset of 100 records to confirm whether the studies indeed covered field work on the species. Based on these methods, we estimate that approximately 2,200 species of mammals have been encountered in the field after 1978 and circa 1,700 over the last 10 years (Table 1). Approximately 1,400 species were included in studies revealing diet information, circa 1,200 species were mentioned in studies collecting fecal samples, and circa 1,600 species were mentioned in trapping studies. Although the opportunities for collection of fecal samples were many, DNA-based evaluation of feces was only published for approximately 550 species (<10% of mammal species). Furthermore, the existing studies examining diet or feeding ecology were heavily skewed toward 64 species that contributed >50% of the records (e.g., wolves, cats, great apes, foxes, and deer), whereas >90% of the species had <10 diet-related records (Fig 1).

**Table 1:** Number of records and species related to keywords examined in *Zoological Record*.

	1978–2018		2009–2018		Estimated % of relevant records/species
Keywords	# records	# species	# records	# species	
Diet	28,106	2,029	9,533	1,451	62/53* 73/70**
Behavior (no diet)	37,133	2,193	12,602	1,592	61/68
Feces	9,995	1,643	4,712	1,228	71/77
Fecal DNA	1,852	730	1,406	666	73/79
Trapping studies	6,584	1,953	2,504	1,303	74/85
Diet + behavior	65,239	2,564	22,135	1,969	67/72
Combined	75,742	3,223	26,977	2,524	65/68

* diet is a major focus of the study.

** study includes field work.

**Fig 2:**
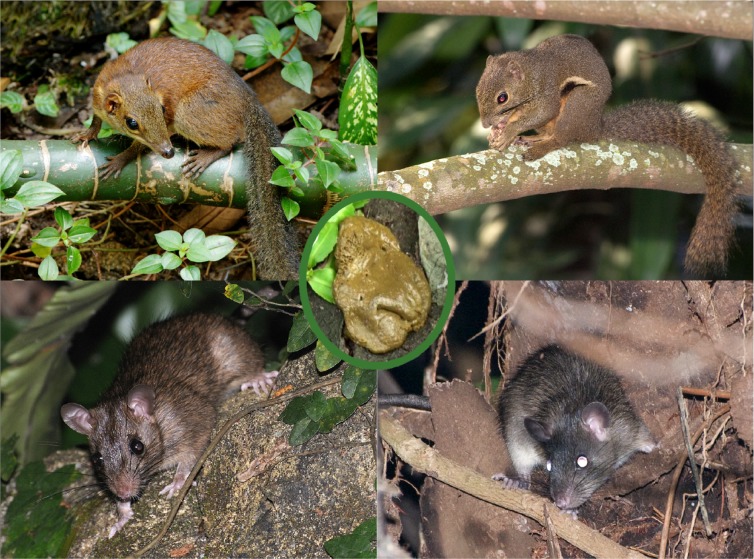
Species trapped in the single night of trapping in Bukit Timah Nature Reserve. Top row: *T*. *glis* (common treeshrew), *C*. *notatus* (plantain squirrel). Bottom row: *R*. *tanezumi* (Asian house rat), *S*. *annandalei* (Annandale’s rat). *Photo credits*: *Nick Baker (mammals) and Andie Ang (fecal sample)*.

**Fig 3:**
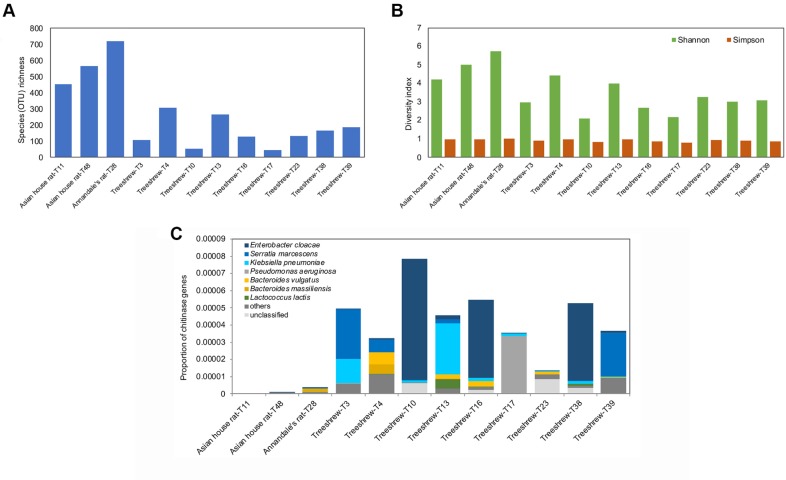
Microbiomes of the small mammals. (A) Microbial OTU richness. (B) Shannon and Simpson diversity indices. (C) Relative abundance of chitinases in gut microbiomes. The data underlying this figure are in S1 Data. OTU, operational taxonomic unit.

In order to test how much natural history information can be obtained during routine field work, we joined a team of mammalogists for a single night of small mammal trapping in one of Alfred Wallace’s favorite collecting sites (Bukit Timah Nature Reserve in Singapore). We sequenced 13 fecal samples from 4 understudied small mammal species collected during that night. Analysis of shotgun sequencing data confirmed many published natural history observations on diet and gut parasites, but the data also provided many new natural history insights. In some cases, the old and new data interacted to provide more in-depth insights. For example, based on published natural history research, treeshrews were known to feed on arthropods and have surprisingly short digestion times (<1 hour), but the shotgun data were needed to reveal that these traits are likely mediated via a microbiome rich in bacteria producing chitinolytic enzymes (Box 1, Figs 2 and 3).

**Box 1. An exploration of fecal samples from a night of trapping**

We joined a survey of small mammals in Singapore’s Bukit Timah Nature Reserve. Small mammals were trapped using banana or papaya as bait, and fecal samples were collected during the 1-night survey. We obtained 23–87 million DNA sequences for 9 samples of the common treeshrew (*Tupaia glis*), 2 samples of the Asian house rat (*Rattus tanezumi*), one sample each of the Annandale’s rat (*Sundamys annandalei*) [31], and a plantain squirrel (*Callosciurus notatus*) (Fig 2). We then used the metagenomic data to reconstruct the mitochondrial genomes for all 4 species. The data revealed that 1 specimen had been incorrectly identified as *S*. *annandalei* although its COI sequence was identical to several *R*. *tanezumi* “R3” records in GenBank (e.g., KC010287, JX533909, HM217503) [32]; i.e., metagenomic data help with validating species identifications and generate a genetic signature for each sample that can be reassigned in case species boundaries change over time.

When we screened the metagenomic data for parasites, numerous nematodes and protists were found to inhabit the guts of treeshrews and rodents; many of these records were new (Table C in S1 Text). This included a potentially new species of *Strongyloides* in treeshrews, which also harbored the protist *Hypotrichomonas*, which was originally described from reptiles and birds but has more recently been found in some mammals [33,34]. Remarkably, the genetic signature (18S rDNA, including hypervariable regions) was identical to a record from an African primate (*Otolemur garnettii*: HQ149966).

We also characterized the diet of the 4 small mammal species. This provided a mixture of novel insights and confirmation of old records (Tables D and E in S1 Text). The treeshrews had fed on many arthropods [35], some of which could be identified to species based on the metagenomic data. A screen of the gut microbes revealed a microbial community that was rich in Proteobacteria such as *Enterobacter*, *Klebsiella*, *Serratia*, and *Pseudomonas* (Table F in S1 Text). Functional profiling revealed high abundance of genes coding for chitinolytic enzymes (Fig 3C); i.e., this gut microbiome may be responsible for the fast digestion of the arthropod-rich diet of treeshrews. Note that they have a remarkably narrow and simple digestive tract with very short food transit times (20–57 minutes) [36–39]. Additional help with digesting arthropods may also come from the treeshrew’s genome given that a close relative (*T*. *belangeri chinensis*) has a larger number of chitinase gene copies as compared with herbivorous and carnivorous mammals (5: [40]). Although the treeshrew microbiomes had a high abundance of chitinolytic bacteria, the overall microbial diversity was low. In contrast, the microbiomes of *Rattus* and *Sundamys* were diverse (1.5–15-fold higher, Fig 3A and 3B) but largely lacking with regard to microbial species producing chitinolytic enzymes.

## It is time for a metagenomic initiative supporting natural history research in the 21st century

Greene (2005) [2] sums up the frustrations of a naturalist by stating “I sat through a symposium on predator–prey interactions, becoming increasingly frustrated as speaker after speaker appealed for ‘more empirical data’ to test the theories on which their talks were focused. In the open discussion that followed, I asked, ‘But who will do that work, and who will pay for it?’” We here argue that existing field work is full of missed opportunities and much natural history data could be obtained through the multidimensional characterization of fecal samples. We also believe that such characterization has the potential to help with raising funds for field work because it can significantly increase the amount of data that can be collected during field work. Fortunately, the cost of “metagenomic clean-ups” is no longer exorbitant. Each sample can now be sequenced for US$200–US$250 (DNA extraction, library preparation, 10 Gbp of sequencing, US$10 for bulk shipping: S1 Text).

But who will do the work, and who will pay for it? Based on our literature search, one could obtain fecal samples for 1,000 mammal species within 10 years. If one were to carry out a metagenomic analysis for 5 samples per species, the sequencing cost for a global initiative would be US$1 million in total, or US$100,000 per year. We estimate that a team of <5 researchers would be able to organize, develop the standard operating procedures (SOPs), and conduct and/or assist with the bioinformatics. The in-depth analysis of the data should be a collaborative effort between field researchers and molecular ecologists with both parties benefiting and contributing complementary expertise. Overall, the cost of such an initiative would thus be significant, but modest when compared with the amount of funding that is currently raised for large-scale barcoding (BIOSCAN: US$180 million). Yet, the benefits would be manifold: it would help with gaining a functional understanding of biodiversity for taxa, such as mammals, in which species diversity and ranges are already comparatively well understood. It would also help with justifying field work and encouraging data collection for noncharismatic species. Lastly, preliminary data obtained through such a project would help with raising funds for more focused projects on species with intriguing preliminary data. For example, the *Tupaia* data presented here may inspire additional work on how microbiomes facilitate the rapid digestion of arthropods.

One concern may be the biased/random nature of the samples that are obtained serendipitously. However, similar to what is the case for traditional natural history data, data for the same species would accumulate over time and be useful for addressing a large number of research questions that are of both academic and applied interest. Here are some examples: Which species share gut parasites? How do parasite loads change across range, population size, and genetic variability? How much variability is there in the microbiomes, and how is it influenced by evolutionary relationships, diet, sex, and age? How many supposedly “phytophagous” species also feed on animals? To what extent is the diet of species determined by evolutionary history? How does the diet change over a species’ range, and how is it related to environmental variables? How do animals in urban areas differ from wild populations?

Of course, metagenomic data are not devoid of problems. This includes false positive “diet” items (flies, dung beetles, and millipedes), which can come from animals that visited the feces (for example, those found in our case study, Table D in S1 Text). An additional issue is that DNA signals from the bait has to be subtracted. Overall, it is thus important to slightly modify existing field protocols by, for example, only using freshly cleaned traps and recovering the samples quickly (Box 2). With regard to benefit sharing and regulatory concerns, we suggest that the extracted DNA should remain with the researchers residing in the country of origin. They would then initiate data acquisition following approved protocols for the country in question (e.g., sequencing within the country or outsourcing). The data—but not the DNA—could then be shared with team members living elsewhere. The cost for sequencing would ideally be borne by a global initiative, but it can also be covered by regional research projects. If organized along these lines, such an initiative would provide benefits for all parties involved. In order to start such an initiative, researchers at the National University of Singapore and the Genome Institute of Singapore will sequence and analyze the first 250 samples (circa 50 species).

**Box 2. Standard operating procedures for collecting fecal DNA**

1. Equipment for field work: Sterilized vials, gloves, and spatula. Ethanol can be added to the vials if field work is extensive and freezer access is limited. For field work involving several days, silica beads can be used for dehydration (see point 4).
2. Equipment for molecular work: Centrifuges and pipettes for multidimensional characterization. When microbiome is the focus, a vortex (Vortex-Genie 2) for bead-beating.
3. Collection of sample: Sample should be collected when defecation is observed or shortly thereafter. If this is not possible, PCR-based screening can be used for species identification. Traps should be cleaned/sterilized between uses.
4. Preservation of sample: Frozen within a few hours or preserved using transport/storage media such as DNA shields (Zymo Research). When such preservation is not possible, a 2-step preservation method can be used: first, storage in ethanol and then desiccation in silica after 1 day [41].
5. Metadata collection: Record location/GPS coordinates, date and time of collection, whether defecation was observed, the exact location of sample (on ground/leaves/inside trap, etc.), type of traps and baits used, and observation of other animals on the feces or bait.
6. Lab work: DNA extraction (QIAGEN DNeasy Blood & Tissue kit) for multidimensional work of this nature, preferably sampling the interior of the sample. The same kit can be used to extract the outer layer if the aim is to maximize host DNA. Kits such as the QIAGEN DNeasy PowerSoil kit if microbiome characterization is the focus.

## Ethics statement

Small mammal trapping was conducted as part of the Bukit Timah Survey initiated by the National Parks Board [42]. Fecal samples were collected after the animals were released from the traps.

## Supporting information

S1 Text Methods and additional detailed results for metagenomic analyses. (DOCX)

S1 Data Data for Figs 1 and 3. (XLSX)
