# Supplementary material for: Boosting natural history research via metagenomic clean-up of crowdsourced feces
Source: PLoS Biol. 2019 Nov 7;17(11):e3000517. doi: 10.1371/journal.pbio.3000517 (PMC6863569; doi:10.1371/journal.pbio.3000517)
Supplement: S1 Text — (DOCX) [file pbio.3000517.s001.docx]

**SUPPLEMENTARY MATERIAL**

This document describes the methods involved in the metagenomic analyses (Section I). It furthermore documents the natural history findings in depth (Section II).

**Section I. METHODS**

**Ethics Statement**

Small mammal trapping was conducted as part of the Bukit Timah Survey initiated by the National Parks Board [1]. Faecal samples were collected after the animals were released from the traps.

**Survey of *Zoological Record***

We downloaded all records under *Zoological Record* available from 1978-2018 using search terms “ST=Mammalia” (supertaxon Mammalia) and limited the search to articles. We then identified all records that contained a full species name also found in the checklist by Burgin et al. (2018) [2] which contains the names for 6399 extant mammal species. We also searched for the genus name and species epithet separately; we included the record if both were present and a manual inspection confirmed its validity (ca. ~300 species). This method was able to identify records using abbreviated genus names (e.g., =*T. glis*) and records pertaining to subspecies that had since been elevated to species. We analyzed all records and a subset covering the last decade.

We next estimated how many records pertained to studies involving field work. This was done by binning the records into four types of studies: (1) feeding ecology and/or diet, (2) behavior, (3) collection of faecal samples and (4) trapping of animals. For (1) and (2), we catalogued all the terms in Zoological Records’ “broad terms” field which provides information on the type of study. Major terms relating to feeding ecology/diet and behavior were then determined (Table A) and records were retrieved and classified by species. However, the species was excluded if a mammalian species was prey and this information was found in the “Organism” field. Records were only retained if the mammal species was the predator. For (3) and (4) “broad terms” could not be used, and we instead used search terms to query the full record (title or abstract or any other field). Lastly we obtained an estimate of (5) studies involving DNA/molecular work on faecal samples by searching title and abstract. Other fields were excluded because terms such as “molecular” and “genetic” are often found in authors’ addresses. Overall, the details of the search terms are in Table A.

Table A: Search terms used for literature survey.

| Category Number | Type of record | Search terms (inclusion) | Search terms (exclusion) |
| --- | --- | --- | --- |
| 1. | Diet/Feeding ecology | Diet  Feeding behaviour  Prey  Predators  Feeding  Carnivorous feeding  Foraging  Predation  Herbivorous feeding | Taxonomy  Systematics  Nomenclature |
| 2. | Behaviour (no diet)^ | Behaviour  Social behaviour  Feeding behaviour  Agonistic behaviour  Group behaviour  Reproductive behavior | Taxonomy  Systematics  Nomenclature |
| 3. | Faecal samples | Feces  Faeces  Fecal  Faecal  Scat  Scats |  |
| 4. | Trapping | Trapping  Trapped  Trap Traps | Camera  Hunting  Poaching  Hunted  Poached |
| 5. | Faecal DNA* | Any term in (3)  DNA  barcod  sequenc  molecul  genom  genetic  microsatellite |  |

^this set was partially overlapping with (1) and overlapping records were excluded.
*partial word searches were allowed but a space was added before sequenc* and both cases were examined where applicable.

In order to estimate how many of these records pertain to field work that encountered the species in question, we examined a random subset of 100 records for each search result. We only considered records relevant if they covered the area of interest (e.g., diet) and field work for free ranging animals. Here, we considered livestock free ranging unless the diet/behavior was modulated as part of the study design. Diet/feeding ecology studies furthermore had to satisfy at least one of the following two criteria: (a) major focus of study was diet and (b) study involved field work. All other records were excluded. These included (1) records pertaining to animals in an artificial setting (e.g. captivity/laboratory/modulated diet). (2) Records reflecting the area of interest but lacking the opportunity to collect faecal samples (e.g. modelling studies, meta-analyses or review articles). Records pertaining to marine mammals were also excluded unless it was explicitly mentioned that scat samples were collected or a carcass was found. (3) Records that were incorrectly classified.

**Sampling and DNA extraction**

Faecal samples were collected during one night of a small mammal survey involving trapping (May 2016). All samples were collected from Sherman traps except one which was collected from the ground below a wire cage trap. In order to allow for multidimensional characterization of samples, DNA was extracted using QIAGEN DNeasy Blood and Tissue kit (QIAGEN, GmbH) as described in Srivathsan *et al.* (2015) [3]. Care was taken to avoid contaminations; i.e., gloves were changed after processing each sample and all procedures were carried out in a biological safety cabinet. The DNA for 13 of the 16 samples was of sufficient quality and quantity (190-2600 ng) for library preparation with NEBNext Ultra II Library Prep kit (insert size of ~350 bp) and subsequent sequencing with Illumina HiSeq 4000 (150 bp PE) at Genome Institute of Singapore.

**Data analyses**

Sequences were trimmed using Trimmomatic v0.33 under the recommended parameters: LEADING:3 TRAILING:3 SLIDINGWINDOW:4:15 MINLEN:50. The ILLUMINACLIP parameter was set to 2:30:10 [4]. Next, we used two approaches for analysing the data. The first was read-based where trimmed reads were directly used to search against databases and the second was assembly-based where reads were first assembled to contigs and scaffolds with subsequent matching of scaffolds to databases. In order to generate assemblies, we used IDBA-UD with multiple k-mer lengths ranging from 20-124 with an identity threshold of 100% [5]. Read-based methods can help detect rare species in metagenomes, but they can lack taxonomic resolution when examined against conserved markers such as rDNA. The latter is less of a concern for assembly-based analyses which, however, tend to overlook rare species in metagenomes [3].

*Identification of plants using chloroplast barcodes*

For plant identifications we relied on read-based identification as developed in Srivathsan *et al*. [3,6]. Here reads were matched against chloroplast markers *rbcL, matK* and *trnL-psbA.* Updated databases of homologous barcode regions were built using publicly available data in GenBank for these markers in a manner similar to Srivathsan *et al*. (2016) [6]. Reads from each dataset were matched against these databases using MEGABLAST and only matches at 98% identity with full length overlaps were retained. BLAST matches were processed using *readsidentifier* v1.1.2 using the single-end criterion which is suitable for 150 bp reads. Only identifications supported by two out of three barcodes were accepted and all identifications supported by only one read were excluded.

*Identification of Metazoa using COI and rDNA*

We used both COI and rDNA databases for identification of Metazoa. For identification based on COI, a database was constructed for Metazoa in a similar manner as described for the plant barcode databases. In addition, we built a second database for all publicly available data for Metazoa in BOLD systems v4. These databases were complemented with COI barcodes sequenced for Singapore specimens [7,8]. Reads were matched against these databases using MEGABLAST setting with e-value 1e-5. Full length matches with >97% identity were classified by the lowest common ancestor algorithm of *readsidentifier*.

Given the lack of representation of many tropical species in COI databases, we also used LSU and SSU rDNA. Given that these markers are generally conserved across multiple species, we (1) only used these genes for order-level identifications and (2) used assembled contigs. The contigs were matched using MEGABLAST to the SSU and LSU rDNA sequences in the publicly available SILVA database (v128) [9]. Only matches having >200bp overlap with reference sequence at an identity >97% were given a taxonomic assignment. All taxonomic assignments were made using *readsidentifier*.

*Identification of parasites*

For characterizing parasites, we also matched the assembled contigs to the Protist Ribosomal reference database (PR^2^) [10] which comprises 18S rDNA sequences and processed the output in the same way as the SSU/LSU rDNA analyses. The results of these analyses suggest presence of *Strongyloides* across multiple species. Fortunately, species-specific diagnostic markers have been developed for identifications using 18S so that they could be used for characterizing the *Strongyloides* matches via alignment of assembled scaffolds to publicly available 18S *Strongyloides* sequences using MAFFT v7 [11]. We then used species-specific diagnostic characters in the three hypervariable regions of 18S as described by Hasegawa *et al.* (2008) [12].

*Characterization of Microbiomes*

For assessing the diversity of gut microbiota, we firstly used RiboTagger to identify 16S sequences from shotgun sequencing data, using only one end of the paired end data [13]. RiboTagger identifies a short recognition sequence (~23 bp long) that is conserved and a hypervariable tag sequence (~33 bp long) that can be used for identification. It was set to retrieve sequences corresponding to v4 region of 16S under --no-eukaryote mode. Diversity indices were calculated using vegan in R [14] and species accumulation curves were plotted using iNEXT [15,16].

Results of RiboTagger suggested poor diversity in the faecal microbiomes of the treeshrews. Given that RiboTagger relies on short “ribotags” from metagenomics reads, OTU (Operational Taxonomic Units) diversity may be underestimated and therefore we next tested if similar patterns were found with full length 16S molecules. Full length 16S were reconstructed using EMIRGE with reference SILVA SSU rDNA database [17]. EMIRGE results in 16S assemblies from shotgun sequence data with abundance information. The resulting 16S molecules were clustered using VSEARCH [18] at 97% similarity with --sizein option to retain the initial abundance estimate using EMIRGE.

Taxonomic classifications were obtained using MetaPhlan2 and both phylum- and species-level classifications were assessed [19]. MetaPhlan2 uses >1 million clade-specific markers obtained from microbial genomes to assess the relative abundance of bacterial species. For functional profiling of the microbiome we used the HMP Unified Metabolic Analysis Network 2 (HUMAnN2) pipeline [20]. HUMAnN2 allows for taxonomically stratified functional profiles of microbiomes by using MetaPhlAn2 for taxonomic profiling, the ChocoPhlAn pangenome database and the UniRef90 database for functional profiling. The abundance of the gene families identified was regrouped based on gene ontology terms and normalized for each sample. The relative abundance values were then compared across samples. Note that for all analyses of microbiomes, we excluded the sample from *Callosciurus notatus* as it was collected from the ground below the trap for the only individual trapped in a wire-cage trap.

*Characterization of host mitochondrial genomes*

One reference mitochondrial genome per host species was assembled using MITObim v.1.9 [21] using a closely related reference genome (*Tupaia belangeri:* AF217811.1*, Rattus tanezumi:* EU273712.1*, Callosciurus erythraeus* NC_025550.1*, Sundamys annandalei* KY464176.1*)*. To assess mitochondrial variability per species, sequences from each metagenomic dataset were mapped onto the reference mitochondrial genome using Bowtie2 [22] with paired-end mode under --end-to-end settings. Samtools [23] was used to process the resulting SAM file and the resulting BAM file was imported into CLC Genomics Workbench (v8) which was used to generate a consensus sequence. For a base to be considered accurately called, the minimum coverage depth was set to 5 with a noise threshold of 0.2. The coverage of host mitochondrial genomes is given in Table B.

**Estimate of sequencing cost**

Our cost estimates are based on sequencing service providers that make the prices public: North Carolina State University (NCSU: <https://research.ncsu.edu/gsl/pricing/>), University of Illinois at Urbana Champaign (UIUC: <https://biotech.illinois.edu/htdna/pricing>), Stanford University (SU: <http://med.stanford.edu/gssc/rates.html>). However, the cost of sequencing for academic purposes tends to be lower than the advertised prices. Our estimates are based on 10 Gbp of data per sample (= 33.33 million 150 bp PE reads). The sequencing price varies depending on the choice of sequencing platform with platform such as NovaSeq 6000 (S4 flowcell) delivering a price of 119 - 123 USD for 10 Gbp. The pricing for library preparation per sample for a mid-scale sequencing project of 24 samples is at 75 - 130 USD. The DNA extraction cost is estimated at ~5 USD, while shipping cost is estimated at 10 USD per samples as long as >20 extractions are sent simultaneously to the nearest sequencing facility. Overall, depending on the strategy and service provider, we estimate the cost of sequencing to be 200-250 USD per sample.

**Section II. Results Tables and Figures**

Table B: Descriptive and assembly statistics for the datasets and host mitochondrial genome coverage. CN: *Callosciurus notatus*, RT: *Rattus tanezumi*, SA: *Sundamys annandalei*, TG: *Tupaia glis*

| Sample ID | Number of raw reads (paired) in millions | Number of reads after trimming (paired) in millions | Number of scaffolds (N50)  in thousands | Average coverage of host mitochondrial genome |
| --- | --- | --- | --- | --- |
| CN-T31 | 75.87 | 67.92 | 450.03 (3852) | 76.46 |
| RT-T11 | 59.48 | 49.22 | 270.44 (2813) | 14.95 |
| RT-T48 | 64.77 | 55.56 | 536.04 (1985) | 32.12 |
| SA-T28 | 66.70 | 58.99 | 735.48 (2171) | 6.43 |
| TG-T3 | 43.49 | 39.56 | 427.58 (1057) | 171.01 |
| TG-T4 | 43.95 | 39.54 | 251.28 (5818) | 13.42 |
| TG-T10 | 56.68 | 50.47 | 55.69 (3460) | 5.05 |
| TG-T13 | 49.16 | 45.05 | 373.45 (1534) | 481.78 |
| TG-T16 | 62.13 | 55.60 | 226.27 (2264) | 152.31 |
| TG-T17 | 37.4 | 32.74 | 45.53 (1831) | 18.73 |
| TG-T23 | 23.12 | 20.79 | 124.68 (2684) | 51.87 |
| TG-T38 | 87.67 | 78.25 | 206.35 (1490) | 12.51 |
| TG-T39 | 32.54 | 29.47 | 155.65 (3008) | 8.23 |

Table C: Gut parasites found in the faecal metagenomes. Presence is highlighted in orange. *CN= Callosciurus notatus, RT= Rattus tanezumi, SA= Sundamys annandalei,* TG= *Tupaia glis.*

|  | **CN-T31** | **RT-T48** | **RT-T11** | | **SA-T28** | **TG-T3** | **TG-T4** | **TG-T13** | **TG-T16** | **TG-T23** | **TG-T39** |
| --- | --- | --- | --- | --- | --- | --- | --- | --- | --- | --- | --- |
| **Nematodes (Rhabdatida)** | | | | | | | | | | | |
| *Nippostrongylus* |  |  |  | |  |  |  |  |  |  |  |
| *Strongyloides* |  |  |  | |  |  |  |  |  |  |  |
| *Travassostrongylus* |  |  |  | |  |  |  |  |  |  |  |
| Unidentified *Rhabditida* |  |  |  | |  |  |  |  |  |  |  |
| **Acanthocephala (Polymorphida)** | | | | | | | | | | | |
| *Centrorhynchus* |  |  |  | |  |  |  |  |  |  |  |
| **Apicomplexa (Eucoccidiorida)** | | | | | | | | | | | |
| *Eimeria* |  |  |  | |  |  |  |  |  |  |  |
| *Isospora* |  |  |  | |  |  |  |  |  |  |  |
| **Metamonada (Diplomonadida)** | | | | | | | | | | | |
| *Spironucleus* |  |  | |  |  |  |  |  |  |  |  |
| *Octomitus* |  |  | |  |  |  |  |  |  |  |  |
| **Metamonada (Tritrichomonadida)** | | | | | | | | | | | |
| *Tritrichomonas* |  |  |  | |  |  |  |  |  |  |  |
| *Simplicimonas* |  |  |  | |  |  |  |  |  |  |  |
| **Metamonada (Trichomonadida)** | | | | | | | | | | | |
| *Tetratrichomonas* |  |  |  | |  |  |  |  |  |  |  |
| *Pentatrichomonas* |  |  |  | |  |  |  |  |  |  |  |
| **Metamonada (Hypotrichomonadida)** | | | | | | | | | | | |
| *Hypotrichomonas* |  |  |  | |  |  |  |  |  |  |  |
| **Others** | | | | | | | | | | | |
| *Blastocystis* |  |  |  | |  |  |  |  |  |  |  |

Table D: Metazoan identifications (bold=order-level identifications that are made by rDNA databases; grey = identifications based on COI, and overlaps represent identifications made by both. Numbers in the brackets represent number of reads matching to COI). TG= *Tupaia glis, CN= Callosciurus notatus, RT= Rattus tanezumi, SA= Sundamys annandalei.*

| **Sample** | **Order** | **Family** | **Species** |
| --- | --- | --- | --- |
| **Common treeshrews (*Tupaia glis)*** | | | |
| TG-T39 | **Hymenoptera (11)** | Formicidae (11) | *Anoplolepis gracilipes* (11) |
|  | Araneae (3) | Araneidae (3) | *Argiope pulchella* (3) |
|  | Lepidoptera (3) |  |  |
|  | **Coleoptera** |  |  |
|  | **Polydesmida** |  |  |
| TG-T38 | **Hymenoptera (10)** | Formicidae (10) | *Polyrhachis proxima* (10) |
|  | **Diptera (10)*** |  |  |
|  | Lepidoptera (4) | NA | *Lepidoptera sp. XSBN* (4) |
|  | **Orthoptera** |  |  |
|  | **Coleoptera** |  |  |
| TG-T23 | Isoptera (9) | Termitidae (9) | *Odontotermes oblongatus* (9) |
|  | **Hemiptera** |  |  |
|  | **Oribatida** |  |  |
| TG-T17 | **Lepidoptera* (24)** | Sphingidae (21) | *Cypa decolor* (20) |
|  |  | Nymphalidae (2) |  |
|  |  |  |  |
| TG-T3 | **Coleoptera** |  |  |
| TG-T10 | **Diptera** |  |  |
| **Plantain Squirrel (*Callosciurus notatus)*** | | | |
| CN-T31 | Lepidoptera (5) | Sphingidae (5) |  |
|  | **Hymenoptera (2)** | Formicidae (2) |  |
|  | Diptera (3) | Cecidiomyidae (3) |  |
|  | **Collembola** |  |  |
| **Annandale’s rat (*Sundamys annandalei)*** | | | |
| SA-T28 | **Isoptera* (40)** | Termitidae (40) | *Odontotermes oblongatus*(28) |
|  | **Coleoptera** |  |  |
| **Asian house rat (*Rattus tanezumi)*** | | | |
| RT-T11 | **Diptera** |  |  |
| RT-T48 | **Diptera (15)** |  |  |
|  | (Gastropoda) (6) | Veronicellidae (6) | *Semperula wallacei* (6) |

*LSU/SSU match was <97%.

Table E: Identifications of plants from the metagenomes in the study. TG= *Tupaia glis, CN= Callosciurus notatus, RT= Rattus tanezumi, SA= Sundamys annandalei.* Values in brackets represent number of matching reads to plant barcodes, i.e. abundance.

| **Sample** | **Family** | **Genus** |
| --- | --- | --- |
| TG-T3 | Araceae (18) |  |
| TG-T39 | Apocynaceae (80) | *Willughbeia* (39) |
| TG-T38 | Dipterocarpaceae (200) | *Shorea* (125) |
|  | Arecaceae (86) |  |
| TG-T23 | Strombosiaceae (5) | *Strombosia* |
| CN-T31 | Fabaceae (55) | *Derris* (23) |
|  | Ixonanthaceae (22) | *Ixonanthes* (22) |
|  | Moraceae (1074) | *Artocarpus* (79) |
|  |  | *Ficus* (23) |
|  |  | *Broussonetia* (55) |
|  |  | *Prainea** (33) |
|  | Myristicaceae (21) |  |
| SA-T28 | Annonaceae (7) |  |
|  | Rosaceae (6) |  |
| RT-T11 | Fabaceae (59) |  |

* *Prainea* were also identified from Moraceae but no clear record of this genus in Singapore. Identification made by more conserved barcodes rbcL and trnL-F but not matK, increasing the probability that it is a misidentification

Table F: Common bacteria present in the various faecal samples. Only species present with >=10% of relative abundance in any of the microbiomes are listed. The relative abundance is colour coded (yellow to green 🡪 low to high), while red signifies absence.

| **Species** | **TG-T13** | **TG-T16** | **TG-T17** | **TG-T23** | **TG-T10** | **TG-T4** | **TG-T3** | **TG-T38** | **TG-T39** |  | **RT-T11** | **SA-T28** | **RatT-T48** |
| --- | --- | --- | --- | --- | --- | --- | --- | --- | --- | --- | --- | --- | --- |
| *Enterobacter cloacae* | 2.4 | 43.9 | 0.1 | 0.3 | 83.3 | 1.4 | 0.5 | 46.7 | 2 |  | 0.2 | 0.1 | 4.1 |
| *Klebsiella pneumoniae* | 26.2 | 1.9 | 2 | 0.3 | 1.6 | 0.7 | 26.6 | 1.8 | 0.3 |  |  |  |  |
| *Escherichia unclassified* | 2.4 | 9.5 | 0.1 | 1.8 | 2.3 | 1.9 | 14.1 | 22.8 | 0.7 |  | 0.2 | 0.4 | 9.5 |
| *Escherichia coli* | 8.8 | 33 |  | 21.9 | 0.01 | 7 | 32.6 | 0.2 | 1.9 |  |  | 2.3 | 18.0 |
| *Morganella morganii* |  | 0.09 | 22.1 | 0.02 | 0.01 | 28 |  | 0.01 | 0.02 |  |  |  |  |
| *Serratia marcescens* | 0.7 | 0.04 |  |  | 0.03 | 3.1 | 16 | 0.04 | 9.2 |  |  | 1.9 |  |
| *Lactococcus garvieae* | 30.8 |  |  | 0.01 |  | 0.35 | 0.1 | 0.1 | 0.2 |  |  |  |  |
| *Pseudomonas aeruginosa* |  |  | 75.1 | 0.2 |  |  | 0.1 | 0.05 |  |  |  |  |  |
| *Weissella cibaria* | 10.4 | 0.01 |  |  |  |  | 0.01 | 2.2 |  |  |  |  |  |
| *Providencia rettgeri* |  |  |  | 0.7 | 0.01 | 0.02 |  |  | 65.5 |  |  |  |  |
| *Clostridium colicanis* |  |  |  | 23.9 |  |  |  |  |  |  |  |  |  |
|  |  |  |  |  |  |  |  |  |  |  |  |  |  |
| *Lactobacillus reuteri* |  |  |  |  |  |  |  |  |  |  | 24.4 | 0.9 | 12.9 |
| *Subdoligranulum unclassified* | 0.02 | 0.01 |  |  |  |  |  |  | 0.04 |  | 3.5 | 12.3 | 0.9 |
| *Parabacteroides unclassified* |  |  |  | 9 |  |  |  |  |  |  | 0.2 | 2.6 | 19.6 |
| *Faecalibacterium prausnitzii* |  |  |  |  |  |  |  |  |  |  | 53.6 | 0.1 |  |
| *Bacteroides massiliensis* | 0.1 |  |  | 1.3 |  | 9.8 | 0.02 |  | 0.2 |  |  | 29.2 |  |

Figure A: Estimation of field work in the various subsets of mammalian records in *Zoological Record* examined. Green (relevant records with field work), Orange (relevant records but for artificial setting), purple (relevant records with no field work), red (not relevant). For diet, an additional category of blue shows records with field work, but less relevance to diet. For combined analyses, all records were considered relevant.

(2)

Figure B: (1) Bacterial OTU richness using EMIRGE and Ribotagger. (2) rarefaction curves for bacterial OTUs based on 16S sequences reconstructed by EMIRGE. Abundance outputs for EMIRGE were rescaled to a million.

Figure C: Relative abundance of bacterial phyla in treeshrew and rodent samples

**REREFENCES**

1. Chan L, Davison GWH. Introduction to the comprehensive biodiversity survey of Bukit Timah Nature Reserve, Singapore, 2014-2018. Gardens’ Bulletin Singapore. 2019;71(Suppl. 1):3-17.
2. Burgin CJ, Colella JP, Kahn PL, Upham NS. How many species of mammals are there? Journal of Mammalogy. 2018;99: 1-14.
3. Srivathsan A, Sha JC, Vogler AP, Meier R. Comparing the effectiveness of metagenomics and metabarcoding for diet analysis of a leaf-feeding monkey (*Pygathrix nemaeus*). Mol Ecol Resour. 2015;15: 250-261.
4. Bogler AM, Lohse M, Usadel B. Trimmomatic: A flexible trimmer for Illumina Sequence Data. Bioinformatics. 2014;30: 2114-2120.
5. Peng Y, Leung HC, Yiu SM, Chin FY. IDBA-UD: a de novo assembler for single-cell and metagenomic sequencing data with highly uneven depth. Bioinformatics. 2012;28: 1420-1428.
6. Srivathsan A, Ang A, Vogler AP, Meier R. Fecal metagenomics for the simultaneous assessment of diet, parasites, and population genetics of an understudied primate. Front Zool. 2016;13: 17.
7. Ho JKL, Foo MS, Yeo D, Meier R. The other 99%: exploring the arthropod species diversity of Bukit Timah Nature Reserve, Singapore. Gardens’ Bulletin Singapore. 2019;71: 391-417.
8. Kutty SN, Wang W, Ang Y, Tay YC, Ho JKI, Meier R. Next-Generation identification tools for Nee Soon freshwater swamp forest, Singapore. Gardens Bulletin Singapore. 2018;70: 155-173.
9. Quast C, Pruesse E, Yilmaz P, et al. The SILVA ribosomal RNA gene database project: improved data processing and web-based tools. Nucleic Acids Res. 2013;41: D590-596.
10. Guillou L, Bachar D, Audic S, et al. The Protist Ribosomal Reference database (PR2): a catalog of unicellular eukaryote small sub-unit rRNA sequences with curated taxonomy. Nucleic Acids Res. 2013;41: D597-604.
11. Katoh K, Kuma Ki, Hiroyuki T, Miyata T. MAFFT Multiple Sequence Alignment Software Version 7: Improvements in performance and usability. Molecular Biology and Evolution. 2005;30: 772-780.
12. Hasegawa H, Hayashida S, Ikeda Y, Sato H. Hyper-variable regions in 18S rDNA of Strongyloides spp. as markers for species-specific diagnosis. Parasitology Research. 2008;104: 869-874.
13. Xie C, Goi CLW, Huson DH, Little PFR, Williams RBH. RiboTagger: fast and unbiased 16S/18S profiling using whole community shotgun metagenomic or metatranscriptome surveys. BMC Bioinformatics. 2015;17: 508.
14. Oksanen J, Blanchet FG, Friendly M, Kindt R, Legendre P, McGlinn D, Minchin PR, O'Hara RB, Simpson GL, Solymos P, Stevens MHH, Szoecs E, Wagner H. vegan: Community Ecology Package. R package version 2.5-5. 2019. Available from: <https://CRAN.R-project.org/package=vegan>.
15. Hsieh, TC, Ma, KH, Chao A. iNEXT: iNterpolation and EXTrapolation for species diversity. R package version 2.0.19. 2019. Available from: http://chao.stat.nthu.edu.tw/blog/software-download/.
16. Chao A, Gotelli NJ, Hsieh TC, Sander EL, Ma KH, Colwell RK, Ellison AM. Rarefaction and extrapolation with Hill numbers: a framework for sampling and estimation in species diversity studies. Ecological Monographs. 2019;84: 45-67.
17. Miller CS, Baker BJ, Thomas BC, Singer SW, Banfield JF. EMIRGE: reconstruction of full-length ribosomal genes from microbial community short read sequencing data. Genome Biol 2011;12: R44.
18. Rognes T, Flouri T, Nichols B, Quince C, Mahe F. VSEARCH: a versatile open source tool for metagenomics. PeerJ. 2016;4: e2584.
19. Truong DT, Franzosa E, Tickle TL, et al. MetaPhlAn2 for enhanced metagenomic taxonomic profiling. Nature Methods. 2015;12: 902-903.
20. Franzosa EA, McIver LJ, Rahnavard G, et al. Species-level functional profiling of metagenomes and metatranscriptomes. Nature Methods. 2018;15: 962-968.
21. Hahn C, Bachman L, Chevreux B. Reconstructing mitochondrial genomes directly from genomic next-generation sequencing reads—a baiting and iterative mapping approach. Nucleic Acids Res. 2013;41: e129.
22. Langmead B, Salzberg SL.Fast gapped-read alignment with Bowtie 2. Nature Methods 2012;9: 357-359.
23. Li H, Handsaker B, Wysoker A, Fennell T, Ruan J, Homer N, marth G, Abecasis G, Durbin R, 1000 Genome Project Data Processing Subgroup. The Sequence Alignment/Map format and SAMtools. Bioinformatics 2009;25: 2078-2079.
